# Nonmuscle Myosin II is Required for Larval Shell Formation in a Patellogastropod

**DOI:** 10.3389/fcell.2022.813741

**Published:** 2022-02-03

**Authors:** Xinyu Liu, Pin Huan, Baozhong Liu

**Affiliations:** ^1^ CAS and Shandong Province Key Laboratory of Experimental Marine Biology, Institute of Oceanology, Center for Ocean Mega-Science, Chinese Academy of Sciences, Qingdao, China; ^2^ Laboratory for Marine Biology and Biotechnology, Pilot National Laboratory for Marine Science and Technology (Qingdao), Qingdao, China; ^3^ University of Chinese Academy of Sciences, Beijing, China

**Keywords:** Lottia goshimai, shell, actomyosin, F-actin, nonmuscle myosin II

## Abstract

The molecular mechanisms underlying larval shell development in mollusks remain largely elusive. We previously found evident filamentous actin (F-actin) aggregations in the developing shell field of the patellogastropod *Lottia goshimai*, indicating roles of actomyosin networks in the process. In the present study, we functionally characterized nonmuscle myosin II (NM II), the key molecule in actomyosin networks, in the larval shell development of *L. goshimai*. Immunostaining revealed general colocalization of phosphorylated NM II and F-actin in the shell field. When inhibiting the phosphorylation of NM II using the specific inhibitor blebbistatin in one- or 2-h periods during shell field morphogenesis (6–8 h post-fertilization, hpf), the larval shell plate was completely lost in the veliger larva (24 hpf). Scanning electron microscopy revealed that the nascent larval shell plate could not be developed in the manipulated larvae (10 hpf). Further investigations revealed that key events in shell field morphogenesis were inhibited by blebbistatin pulses, including invagination of the shell field and cell shape changes and cell rearrangements during shell field morphogenesis. These factors caused the changed morphology of the shell field, despite the roughly retained “rosette” organization. To explore whether the specification of related cells was affected by blebbistatin treatments, we investigated the expression of four potential shell formation genes (*bmp2/4*, *gata2/3*, *hox1* and *engrailed*). The four genes did not show evident changes in expression level, indicating unaffected cell specification in the shell field, while the gene expression patterns showed variations according to the altered morphology of the shell field. Together, our results reveal that NM II contributes to the morphogenesis of the shell field and is crucial for the formation of the larval shell plate in *L. goshimai*. These results add to the knowledge of the mechanisms of molluskan shell development.

## Introduction

Mollusks develop their shells at early developmental stages. In most molluskan lineages (conchiferans), a shell plate can be observed shortly after the completion of gastrulation (e.g., in trochophores) on the dorsal side of the embryo/larva. The nascent shell plates show high similarities among conchiferan clades, such as bivalves and gastropods (Bielefeld and becker, 1991; Mouëza et al., 2006), which comprise the major molluskan clades, and scaphopods (Wanninger and Haszprunar, 2001), which is a much smaller clade, but its members also have trochophore larvae. In previous studies, different terms have been used to refer to the tissue that develops during embryogenesis and subsequently secretes the larval shell plate. As mentioned previously (Yang et al., 2020), here, we use the term “shell field” to refer to this tissue, which we think will facilitate interspecies comparisons.

The shell fields of conchiferans typically exhibit concentric organizations, i.e., a “rosette” pattern, with different types of cells arranged in varied distances with respect to its center (Kniprath, 1981). The morphogenesis of the shell field represents an essential phase of shell development and thus has been investigated in various gastropod and bivalve lineages (Kniprath, 1980; Eyster, 1983; Mouëza et al., 2006; Silberfeld and Gros, 2006; Hohagen and Jackson, 2013). Despite the considerable interspecies variations, a common developmental process of the shell field is that it invaginates in the early developmental phase (Kniprath, 1981). Such invagination can generate a pore on the dorsal side that can be much wider than the blastopore on the ventral side, forming a prominent feature of molluskan embryos (Tomlinson, 1987; Mouëza et al., 2006; Tan et al., 2017). In some species, the margin tissues seal the invaginated shell field to form a closed lumen (Eyster, 1983; Hohagen and Jackson, 2013). Given the common invagination process during shell field morphogenesis, it can be expected that it may have existed in the common molluskan (conchiferan) ancestor. The mechanisms underlying this process thus may contribute to understanding the origin and evolution of molluskan shells, yet they remain largely unclear.

We recently investigated shell development in the patellogastropod *Lottia goshimai*, which is phylogenetically distant from many other gastropods (e.g., *Lymnaea* or *Ilyanassa*). The shell field morphogenesis of *L. goshimai* shows quite different characteristics compared with those of other gastropods, such as *Lymnaea* (Hohagen and Jackson, 2013) and *Aeolidia* (Eyster, 1983). For instance, its shell field only exhibits slight invagination and shows constant contact with meso/endodermal tissues during morphogenesis (Yang et al., 2020). Moreover, we found that filamentous actin (F-actin) was aggregated in the shell field of *L. goshimai* (Yang et al., 2020). To the best of our knowledge, this phenomenon has not been reported in other molluskan lineages.

**FIGURE 1:**
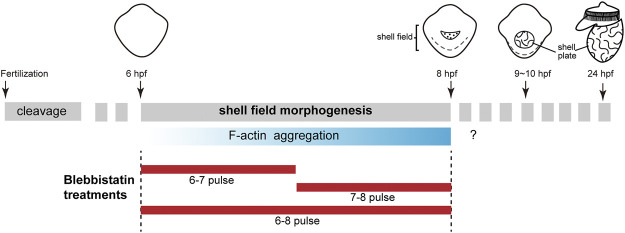
F-actin aggregates in the shell field during the early development of *L. goshimai*. The upper row shows the general development of *L. goshimai*, with the key developmental events of shell development emphasized. Shell field morphogenesis could be morphologically detected after 6 hpf and generally finished at 8 hpf. The shell plate could be observed since 9 hpf, and the 24-hpf veliger larva exhibited well-developed shell plate characteristics in gastropods. Thus, the period from 6 to 8 hpf should be the key stage for the morphogenesis of the shell field. We found that F-actin gradually aggregated in the shell field during this period (lower row). The question marks indicate that we did not determine whether F-actin aggregations were detectable in subsequent developmental stages. The design of the blebbistatin treatment experiments is shown below, which includes pulses in three time windows.

In *L. goshimai*, F-actin starts its aggregation 6 h post-fertilization (hpf) (Figure 1). Coincidently, this period is also the stage when the shell field starts to show evident morphogenetic changes (invagination). Moreover, the central region of the shell field, which shows the strongest F-actin aggregation, exhibits maximum invagination (Yang et al., 2020). Thus, F-actin aggregation and invagination of the shell field exhibit temporal and spatial correlations, which strongly suggests that F-actin participates in the invagination of the shell field, possibly by mediating a force-driven process. Generally, F-actin-mediated force-driven processes involve nonmuscle myosin II (NM II), which supplies energy for mechanical forces, and the two molecules together form actomyosin networks (Jacinto and Baum, 2003; Agarwal and Zaidel-Bar, 2019). Based on this idea, in the present study, we explored the functions of NM II in the shell development of *L. goshimai*. The results supported the crucial role of NM II (actomyosin networks) in the morphogenesis of the shell field as well as in the formation of larval shell plates in *L. goshimai*.

## Materials and Methods

### Animals

Adult *L. goshimai* Nakayama, Sasaki & Nakano, 2017, were collected from intertidal rocks in Qingdao, China. Gamete collection and artificial fertilization were performed as previously described (Huan et al., 2020). Fertilized eggs were cultured in filtered seawater (FSW) containing antibiotics (100 unit/mL benzylpenicillin and 200 μg/ml streptomycin sulfate) in an incubator at 25°C.

### Drug Treatment

Blebbistatin (Selleckchem, S7099) was dissolved in dimethyl sulfoxide (DMSO) at a concentration of 10 mM to prepare the storage solution, and it was stored at −80°C. Drug treatment experiments were performed using 6-well plates. At the desired developmental stage (6 or 7 hpf), the storage blebbistatin solution was added to the seawater to a final concentration of 15 μM. After one or 2 hours, the blebbistatin was removed from the culture system by washing with FSW at least three times, and the embryos were fixed immediately or raised to 10 or 24 hpf. In total, three types of treatments were performed (6-7, 7-8 and 6-8 pulses; see Figure 1). Equivalent volumes of DMSO were added to the seawater in the control groups, and the same treatment time windows were used. Three batches of embryos derived from different female parents were used for every treatment to ensure reproducibility.

Sample fixations were conducted as previously described with minor modifications (Koop et al., 2007; Fritsch et al., 2016; Kristof et al., 2016; Henry et al., 2017). In brief, samples for immunostaining, phalloidin staining and whole mount *in situ* hybridization (WMISH) were fixed with 4% paraformaldehyde (PFA) (1 × PBS, 100 mM EDTA, 0.1% Tween-20, pH 7.4) overnight at 4°C. For scanning electron microscopy (SEM), the samples were fixed in 2.5% glutaraldehyde (diluted in 1 × PBS with 0.1% Tween-20) overnight at 4°C. To explore larval shell development, the 24-hpf larvae were relaxed in 125 mM magnesium chloride and then fixed in 2.5% glutaraldehyde for 2 h at room temperature. After fixation, the samples were washed several times with PBST (1 × PBS with 0.1% Tween-20) and stored at 4°C until use. For WMISH, the samples were dehydrated to 100% methanol and stored at −20°C.

### Immunostaining and Phalloidin Staining

A primary antibody (Cell Signaling, 3671S) was used to recognize the conserved phosphorylated residues (Ser19) of the light chain of NM II. This antibody has been shown to stain phosphorylated NM II (pNM II) in mollusks (Toledo-Jacobo et al., 2019). For immunostaining, samples were first permeabilized with 1% Triton X-100 (in 1 × PBS) for 10 min and then treated with 0.05% trypsin (in PBST) for 10 min at room temperature (RT). After washing with PBST, the specimens were blocked in blocking solution (0.5% blocking reagent (Roche) in PBST) for 1 h at RT. Samples were then incubated with blocking solution containing the primary antibody (1:100) and tetramethylrhodamine-labeled phalloidin (Solarbio, CA1610, 1:200) overnight at 4°C. Stained samples were washed five times in PBST at 10-min intervals and then incubated with Alexa Fluor 488-coupled goat anti-rabbit secondary antibody (Proteintech, SA00013-2, 1:200 in PBST) for 1 h at RT. Samples were rotated for incubation with the blocking solution or antibodies.

### WMISH, Scanning Electron Microscopy and Imaging

Samples for SEM were dehydrated to ethanol, followed by a previous procedure before being submitted to SEM (Tan et al., 2017). SEM was performed under a Hitachi S-3400N scanning electron microscope. WMISH was performed as described previously (Huan et al., 2020); the sequences of the primers are provided in Supplementary Table S1. WMISH and immunostaining samples were mounted in 90% glycerol and observed under a Nikon 80i microscope or a ZEISS LSM 710 laser-scanning confocal microscope. In particular, the manipulated 24-hpf larvae were recorded under DIC (differential interference contrast) mode of a Nikon 80i microscope. The parameters of DIC microscopy were carefully adjusted to show the birefringent materials that could reflect the nature of shell materials.

### Reproducibility

In the immunostaining assay performed in normal embryos, three different batches of embryos were used, and at least 20 individuals of each batch were investigated to ensure that they showed consistent results. For the blebbistatin-treated samples, in each phalloidin staining, WMISH and immunostaining assay, the distributions of signals were highly consistent in the manipulated or control group and were evidently different between groups. Thus, the results were determined by visual inspections, and the numbers of individuals were recorded (shown in Figures 5–7). This strategy was also applied in the assay to assess larval shell development in 24-hpf veliger larvae (Figure 3).

For the SEM experiments, since the samples could not be rotated during observations, the investigation of each individual was not applicable. Given that the orientations of the samples varied under SEM, we could only record the samples for which the dorsal side could be discriminated. We confirmed that at least five larvae showed discriminable shell fields in each trial, and their phenotypes were highly consistent.

## Results

### Phosphorylated Nonmuscle Myosin II Colocalized With Aggregated F-Actin in the Shell Field

**FIGURE 2:**
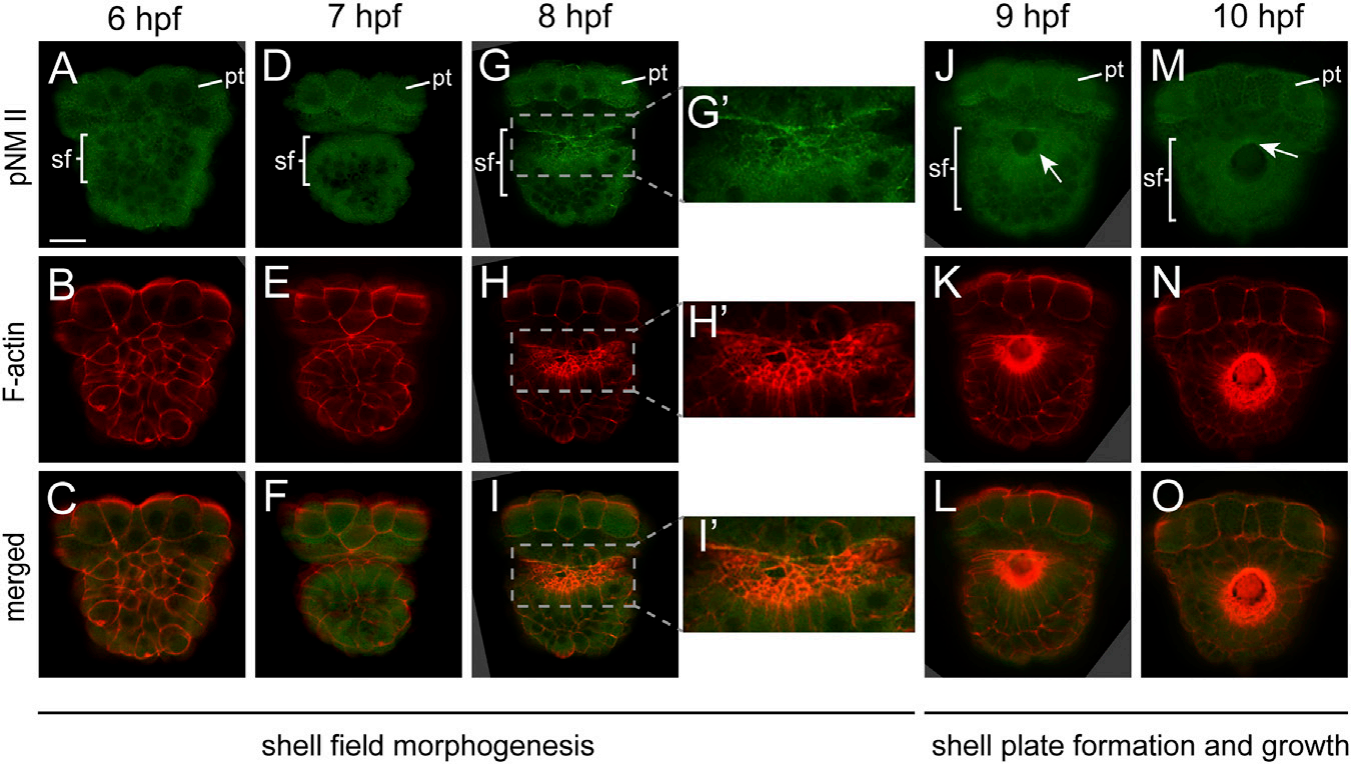
Distribution of pNM II and F-actin during larval shell development. The pNM II was detected using a specific antibody. Its signals could be best discerned at 8 hpf, which showed high degrees of colocalization with F-actin. When the shell plate was formed, the resolution was decreased, but the aggregation of both molecules in the shell plate region was supported (the white arrows in **J** and **M** indicate aggregated pNM II). Panels **G’–I’** show magnified images of the areas that are indicated by dashed rectangles in **G–I**. pt, prototroch; sf, shell field. The bar represents 25 μm.

NM II is phosphorylated for activation. Thus, to explore whether NM II is involved in shell development, we first investigated the distributions of pNM II. In normal development, morphogenesis of the shell field starts before 7 hpf and is generally completed at 8 hpf; the nascent shell plate emerges beginning at 9 hpf (Figure 1). Therefore, we focused on the period from 6 to 10 hpf. The results revealed aggregations of pNM II in the shell field beginning at 8 hpf. In particular, in 8-hpf embryos in which the clearest pNM II signals could be discriminated, it was distributed in the cortical region of shell field cells (Figure 2G,G’). This distribution pattern was highly similar to that of F-actin (Figures 2H,H’,I,I’). At earlier stages (6-7 hpf), although aggregations of F-actin could be recognizable (Figures 2B,C,E,F), those of pNM II were not detectable (Figures 2A,D). This difference may be caused by the relatively low immunostaining signals of pNM II and relatively high fluorescence levels in the cytoplasm compared with those of F-actin. The fluorescence in the cytoplasm after pNM II staining was likely caused by the binding of the primary antibody in the region, which could be unspecific binding, or the binding to the pNM II in intracellular vehicles (e.g., Miklavc et al., 2012; Bedi et al., 2017), and we found it could not be further reduced after trying several available procedures. When the shell plate was developed at 9 and 10 hpf, aggregations of both F-actin and pNM II could also be detected in the shell field (i.e., the larval mantle, with stronger signals in the peripheral region; Figures 2J–O), despite the relatively low resolutions.

### Blebbistatin Pulses Prevented the Formation of Larval Shell Plates

We next investigated the roles of NM II by treating the embryos with the specific inhibitor blebbistatin. This small molecule drug (Straight et al., 2003) has been widely used to inhibit the functions of NM II in animals (Lyons and Weisblat, 2009; Dong et al., 2011). Since morphogenesis of the shell field occurred between 6 and 8 hpf, we focused on this stage in the drug treatment experiments. Specifically, pulse experiments in one- or 2-h periods were performed, namely, 6-7, 7-8, and 6-8 hpf pulses (Figure 1).

**FIGURE 3:**
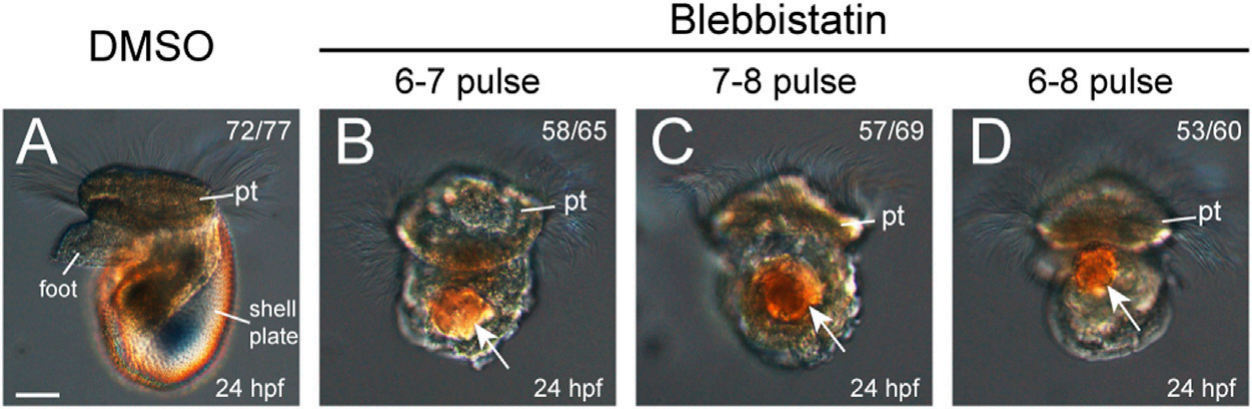
Larval phenotypes (24 hpf) after blebbistatin treatments. All panels show images captured under DIC microscopy, with lateral views anterior to the top. DMSO treatment in any time window did not generate detectable changes in development, and thus, the representative results from one group are shown **(A)**. Larval shell plates were not observed in the manipulated larvae, which only showed birefringence materials (white arrows in **B–D**). pt, prototroch. The bar represents 25 μm.

We first investigated the development of larval shells in 24-hpf veliger larvae, given that they exhibited the most characteristic larval shell plate. As shown in Figure 3, the control veliger larvae exhibited a well-developed shell plate that could encompass the whole larval body (Figure 3A). However, the manipulated larvae of any group did not exhibit a recognizable shell plate (Figures 3B–D). Most of these larvae possessed birefringent materials, which were likely residual shell materials (Figures 3B–D). These birefringent materials may have common chemical compositions comparable to a normal shell plate. However, the correct organization is crucial for a shell plate from a biological perspective. We considered that a molluskan larval shell plate should have a determined shape, itself composed of a relatively thin layer of mineralized materials. We therefore did not recognize the somewhat irregularly shaped, birefringent masses in the treated larvae to be a certain type of shell plate.

**FIGURE 4:**
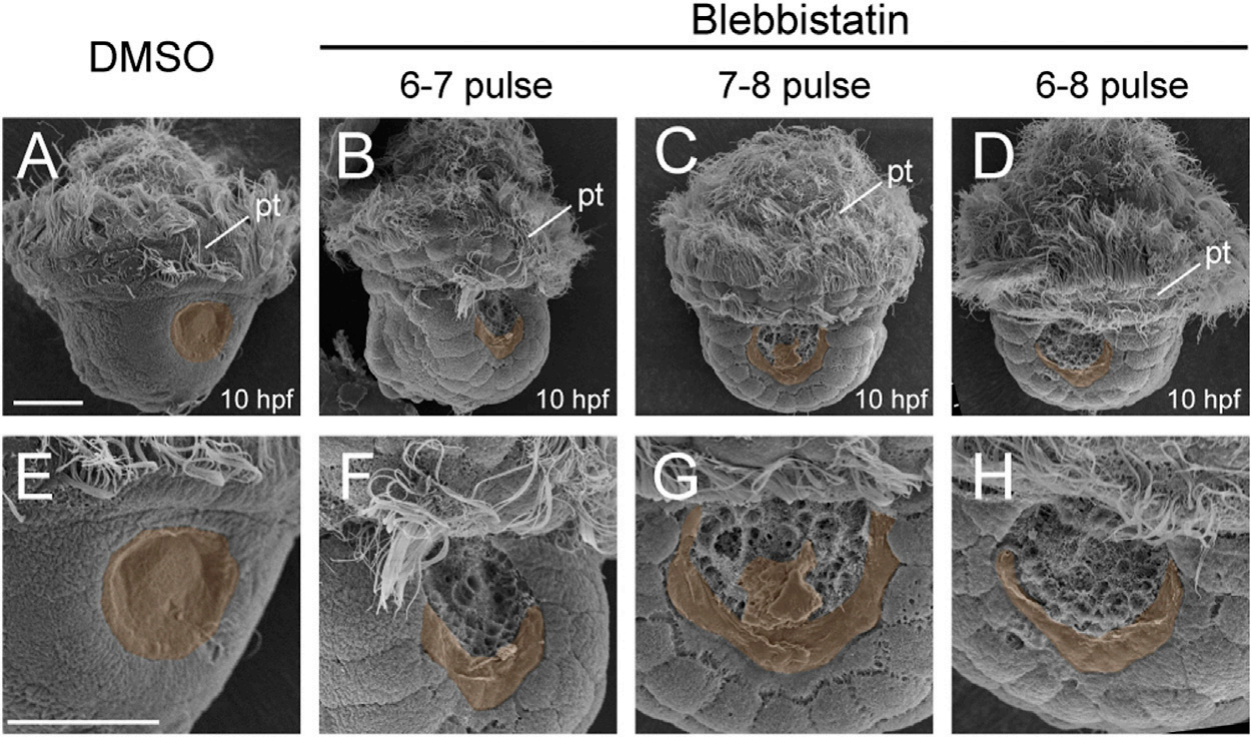
Shell development at 10 hpf after blebbistatin treatments. All panels show SEM images, with dorsal views anterior to the top. Panels **(E–H)** show magnified images to view the details of the shell field. The discriminable shell plates [in **(A)** and **(E)**] or shell pieces due to manipulations [in **(B–D)** and **(F–H)**] are highlighted with brown shadows. pt, prototroch. The bars represent 25 μm.

To explore more details, we investigated an earlier developmental stage, i.e., 10 hpf. At this stage, the control larvae developed a nascent shell plate covering the center of the shell field (Figures 4A,E). In contrast, only a residual shell piece could be observed at the posterior margin of the shell field in the manipulated larvae (Figures 4B–D and Figures 4F–H). These shell pieces were likely composed of a thin layer of shell materials comparable to the normal nascent shell plate, but they were incomplete and could not cover the central region of the shell field that exhibited surface protrusions (compare Figures 4E–H). Given that the normal shell plate began its formation at the posterior margin of the shell field (Yang et al., 2020), these results seem to indicate that shell formation in the manipulated larvae was disturbed since the early phases of its formation.

### Changes in the Shell Field Morphology After Blebbistatin Treatment

**FIGURE 5:**
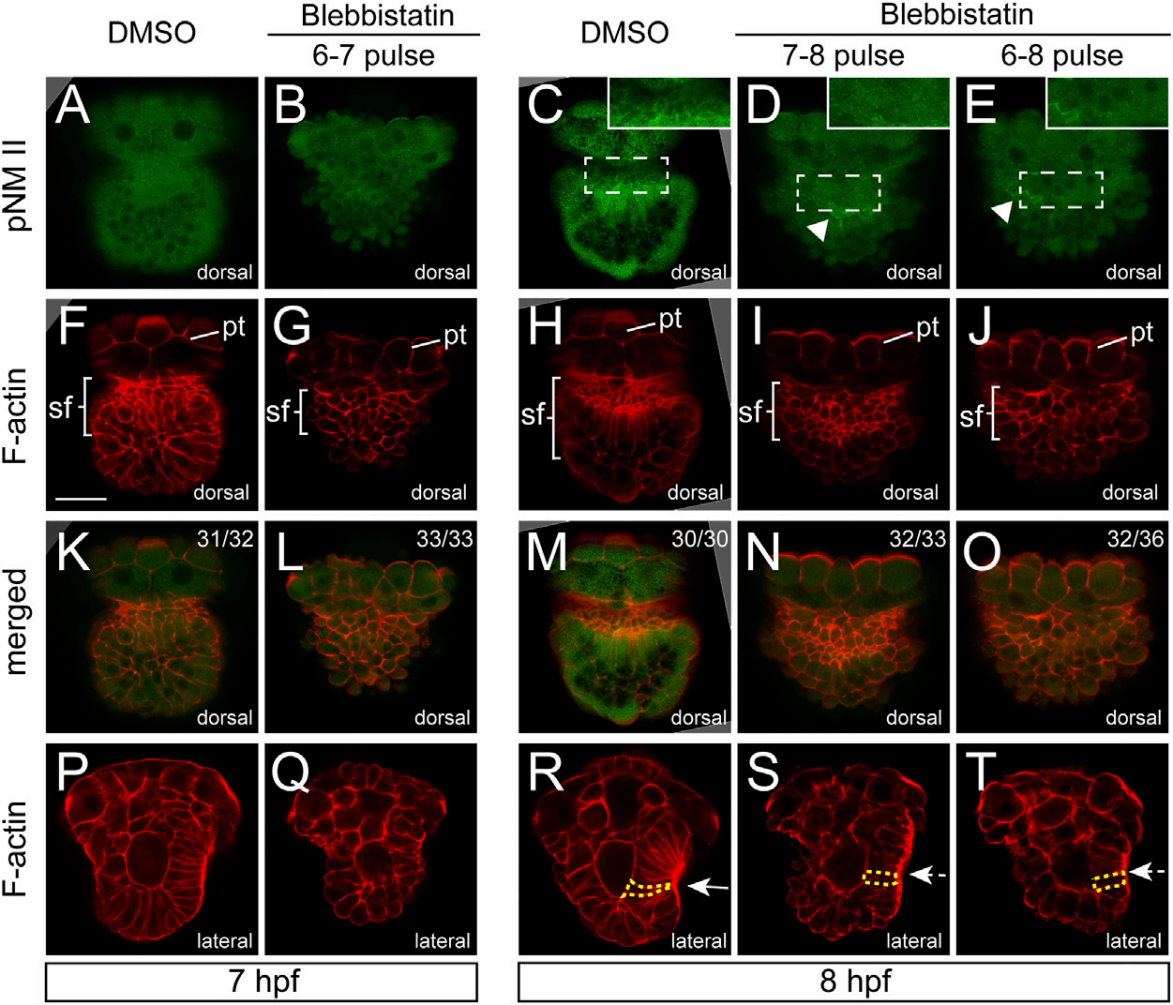
Aggregations of F-actin and pNM II were inhibited by blebbistatin treatments. Panels **(A–O)** show dorsal views, anterior to the top. Generally, aggregations of F-actin and pNM II were inhibited by blebbistatin treatments. The arrowheads in **(D,E)** indicate residual pNM II signals. A linear intensity profile for each labeled region in panels **(C–E)** is provided in Supplementary Figure S1. Panels **(P–T)** show lateral views anterior to the top and dorsal to the right. The invagination of the shell field is evident in control embryos [the arrow in **(R)**] but is inhibited in manipulated embryos [dashed arrows in **(S,T)**]. One representative cell of each group is outlined by yellow dashed lines to show the differences in the cell shape **(R–T)** pt, prototroch. The bar represents 25 μm.

We checked the distributions of F-actin and pNM II after blebbistatin treatments. pNM II was largely eliminated after the treatment (Figures 5A–E), indicating successful inhibition of NM II phosphorylation. We found that the aggregation of F-actin was also greatly reduced. At 7 hpf, F-actin in the normal shell field showed a trend of aggregation (Figures 5F,K), but this trend was not observed in the embryos treated with blebbistatin from 6 to 7 hpf (Figures 5G,L). At 8 hpf, the aggregation of F-actin was particularly evident in control embryos (Figures 5H,M). However, this aggregation was strongly inhibited in the embryos treated with blebbistatin beginning at 6 hpf (Figures 5J,O). In the 7-8 pulse group, F-actin staining in the presumptive shell field was still detectable but showed an evident reduction (Figures 5I,N).

F-actin staining further revealed changes in the organization of the shell field. At 7 hpf, no obvious changes were observed in the shape of shell field cells in manipulated embryos (Figure 5P,Q). At 8 hpf, the normal shell field in the control groups showed evident invagination at the central region (white arrow in Figure 5R), and the cells generally elongated and showed a flask shape (Figure 5R). In contrast, the shell field in the manipulated embryos (for both the 7-8 and 6-8 pulse groups) no longer invaginated (white dashed arrows in Figures 5S,T), suggesting that the invagination of the shell field was inhibited by the blebbistatin treatments. The cells of the shell field also generally showed a column shape (Figures 5S,T), which was very different from the flask-shaped cells in control embryos (Figure 5R). In addition, although we did not quantify it, the elongation of the shell field cells seemed to also be inhibited (compare the labeled cells in Figures 5S,T to that in Figure 5R).

### The Shell Field Showed Altered Organization, but the Cell Specification Was Not Greatly Disrupted

**FIGURE 6:**
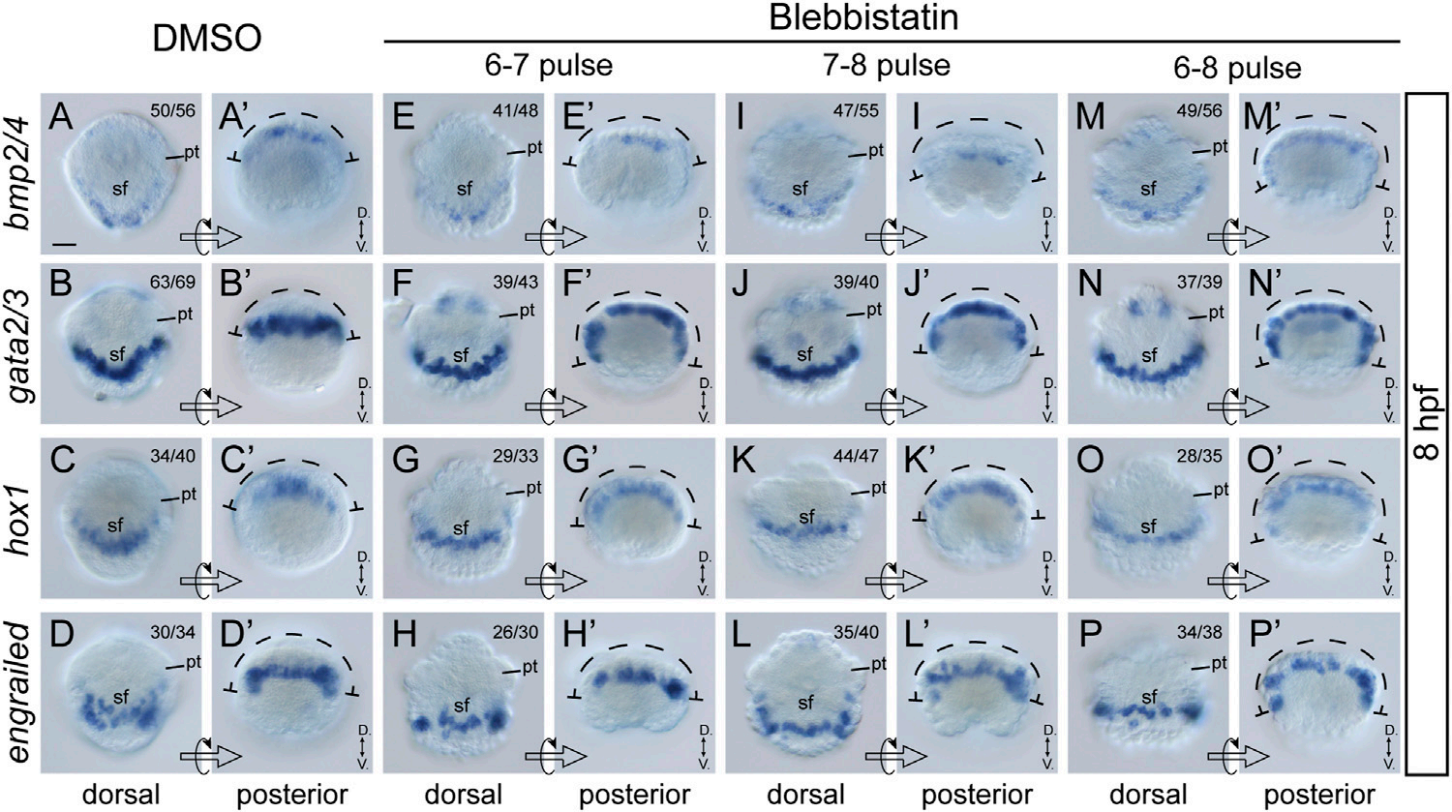
Expression of four pSF genes at 8 hpf after blebbistatin treatments. Panels **(A–P)** show dorsal views, anterior to the top, and **(A′–P′)** show posterior views dorsal to the top. The dashed curves in **(A′–P′)** indicate the width of gene expression regions, which is generally spread in manipulated embryos. D., Dorsal. V., Ventral. pt, prototroch. sf, shell field. The bar represents 25 μm.

**FIGURE 7:**
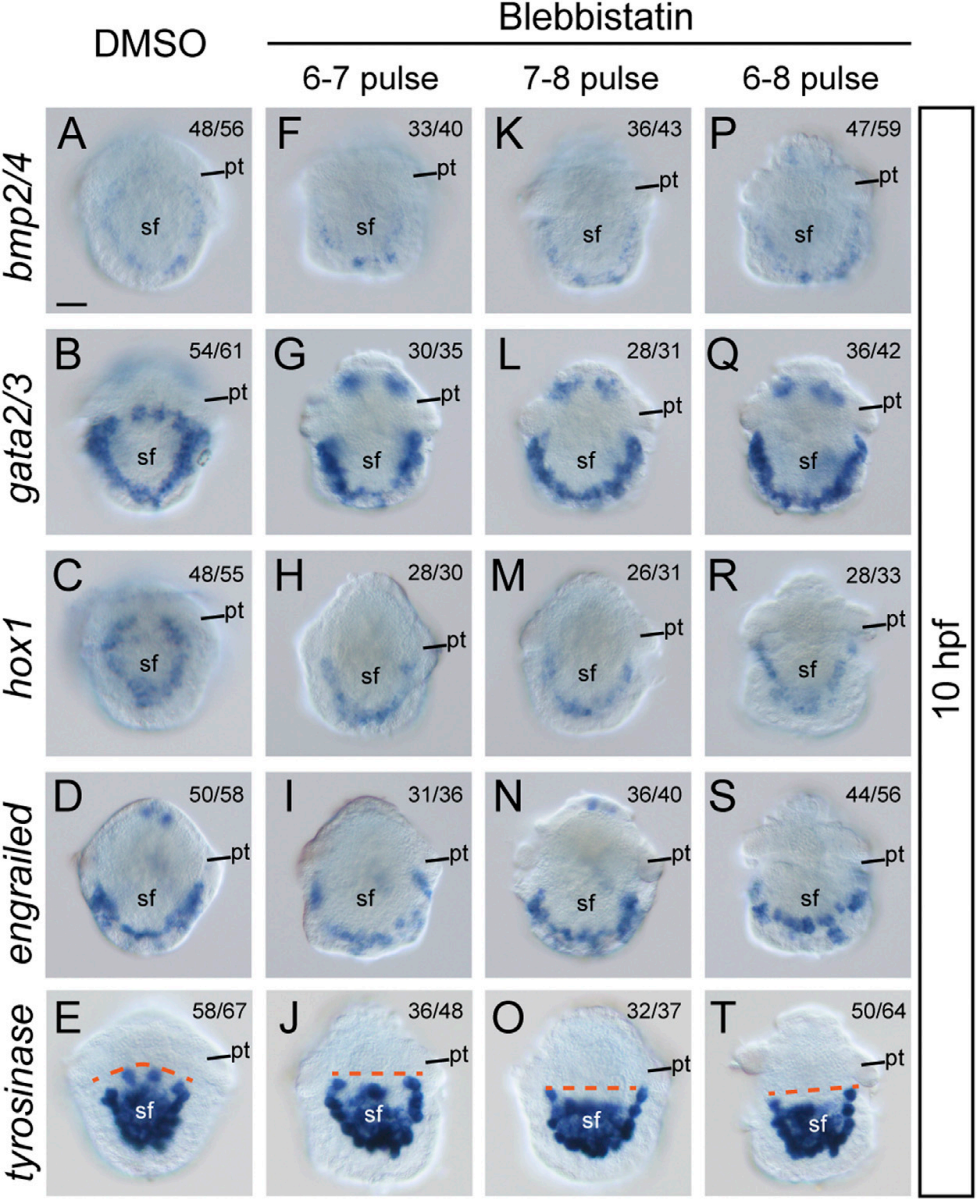
Expression of five pSF genes at 10 hpf after blebbistatin treatments. All panels show dorsal views, anterior to the top. Dashed lines in **E**, **J**, **O**, and **T** indicate the anterior edge of the *tyrosinase* expression regions. pt, prototroch. sf, shell field. The bar represents 25 μm.

Although the most common role of actomyosin networks in animal development is to mediate morphogenetic changes, such as cell shape changes, the mechanical force they generate or propagate may generate additional effects on the cell (Heisenberg and Bellaiche, 2013). For instance, NM II can affect the specification of neural crest cells (Kim et al., 2015). Given that the formation of larval shell plates was seriously interrupted by blebbistatin treatments, we investigated whether the specification of shell field cells was affected by exploring the expression of related genes. Four potential shell-formation (pSF) genes that showed specific expression in the shell field were used, including *bmp2/4*, *gata2/3*, *engrailed* and *hox1* (Yang et al., 2020). Although the specific roles of these genes in shell development are unknown, we proposed that their expression might reflect the specification of related cells. The results revealed comparable expression levels of the four genes between control and manipulated embryos. This was observed either at 8 hpf, when the morphogenesis of the shell field was generally completed, or at 10 hpf, when the shell plate was formed (Figure 6, Figure 7). Conversely, we found that the expression patterns of the genes were substantially different between the control and manipulated samples. At 8 hpf, the expression of the four genes in control embryos showed a generally U-shaped pattern (Figures 6A–D); however, that in the manipulated larvae exhibited a relatively linear pattern (Figures 6E–P). From the posterior view, the gene expression after blebbistatin treatments mostly spread to lateral tissues (Figures 6E’–P’), in contrast to that in control embryos that was restricted to dorsal tissues (Figures 6A’–D’). At 10 hpf, the expression of *bmp2/4*, *gata2/3* and *hox1* exhibited an imaginary circle in control larvae (Figures 7A–C), outlining the shell plate that had been developed during this stage (Figure 4A). In the manipulated larvae, in contrast, the expression of the genes showed a U-shaped pattern (Figures 7F–H,K-M,P–R) but never succeeded in forming a circular pattern. As a sole exception, the expression of *engrailed* did not show a circular pattern at 10 hpf, and we did not detect evident changes in *engrailed* expression patterns between control and manipulated larvae (Figures 7D, I, N, S).

Given that the four pSF genes mentioned above may not be directly involved in shell material secretion, we further investigated a gene that might be involved in shell biogenesis, a *tyrosinase* gene. This gene was suggested to be a biogenesis gene of larval shell in the oyster *Crassostrea gigas* (Huan et al., 2013), and similar roles in *L. goshimai* have been supported with comparable expression patterns (Tan et al., 2018). We found that *L. goshimai tyrosinase* showed comparable expression levels at 10 hpf between manipulated and control larvae (Figures 7E,J,O,T), seeming to indicate that the capacity to secrete shell materials was not inhibited by blebbistatin. However, similar to the other four pSF genes, *tyrosinase* expression patterns changed after blebbistatin treatment. Specifically, the anterior edge of its expression regions became linear in manipulated larvae, especially in the treatments that were terminated at 8 hpf (dashed lines in Figures 7E,J,O,T).

## Discussion

Despite extensive investigations on the development of molluskan shell fields (Kniprath, 1981), the involvement of F-actin has not been reported previously. Conversely, given the common invagination process during shell field morphogenesis in many mollusks (Kniprath, 1981) and the common roles of actomyosin networks in tissue morphogenesis (Jacinto and Baum, 2003; Vicente-Manzanares et al., 2009; Heissler and Manstein, 2013; Agarwal and Zaidel-Bar, 2019), it is not surprising to observe the aggregations of F-actin and to speculate the involvement of the actomyosin networks in molluskan shell development. In the present study, we focused on NM II, which is the core molecule in actomyosin networks, in early molluskan shell development. As expected, our results revealed aggregations of pNM II in the shell field, which largely colocalized with F-actin when discriminable (at 8 hpf, Figure 2). Similar colocalization of the two molecules was also observed in various force-driven processes, such as the compartment boundaries in the wing disc of *Drosophila* and the notochord cells of *Ciona* during tubulogenesis (Major and Irvine, 2006; Dong et al., 2011), supporting the idea that shell field morphogenesis involves force-driven processes mediated by actomyosin networks. This notion was subsequently supported by functional experiments using blebbistatin, a specific inhibitor of NM II. Given that we detected tight correlations between F-actin and pNM II (in both normal and manipulated embryos), in the subsequent text, we will consider blebbistatin-treated experiments to indicate the roles of not only NM II but also the actomyosin networks when this generalization is necessary.

### NM II Functions in Shell Field Morphogenesis but Seemingly Does Not Regulate Cell Specification

Invagination in the early phase of shell field development has been revealed in various mollusks (Kniprath, 1981). However, even though it has been suggested that endodermal tissues induce invagination in some species [such as *Lymnaea* (Hohagen and jackson, 2013)], to the best of our knowledge, there are no reports aiming to decipher the molecular mechanisms underlying this process. We found that the invagination of the shell field was inhibited by blebbistatin treatments, confirming the role of NM II in this process. This result would help to promote the in-depth understanding of this common process of molluskan shell field development (see below).

Our previous results indicate that the shell field morphogenesis of *L. goshimai* involves extensive morphogenetic changes, such as “cell movements” and cell shape changes (Yang et al., 2020). The shape changes of shell field cells include mainly elongation along the apical-basal axis and contractions at the apical side, together transforming the column cells into a flask shape (Yang et al., 2020). After blebbistatin treatments, we found that the cells remained column-shaped and likely no longer elongated, as in control embryos, which suggested that the cell shape changes during normal shell field morphogenesis were mediated by NM II or actomyosin networks. Given that cell shape changes occur at the single-cell level, they may involve asymmetrical distributions of actomyosin networks inside a cell (Agarwal and Zaidel-Bar, 2019). Indeed, we detected asymmetrical F-actin distribution along the apical-basal axis of the shell field cells (Figure 5R). However, comparable asymmetrical distribution patterns were not detected for pNM II, potentially because of the relatively low immunostaining signals of pNM II and the relatively high background fluorescence; improved immunostaining procedures may aid in explorations of whether pNM II is asymmetrically distributed at the subcellular level.

“Cell movements” during shell field morphogenesis refer to the relative changes in the location of shell field cells from the ventral and lateral sides to the dorsal side, as indicated by the continuous changes in pSF gene expression regions (Yang et al., 2020). Recently, we found that these changes in cellular locations should be attributed to complicated morphogenetic changes that include not only cell movements but also other types of developmental events (e.g., axial elongation) (Tan et al., 2021). Thus, we think “cell rearrangement” should be a more precise term to refer to this process. After blebbistatin treatments, we found that the expression of the four pSF genes at 8 hpf resembled that in an earlier normal embryo, characteristic of gene expression regions spreading to lateral tissues and the dorsal expression exhibiting a relatively straight pattern. These results indicated that the blebbistatin treatments inhibited cell rearrangements during shell field morphogenesis and thus revealed the roles of NM II in this process.

Altogether, our results indicate that NM II (actomyosin networks) mediates the major morphogenetic changes during shell field development in *L. goshimai*, including invagination of the shell field, cell shape changes and cell rearrangements, thereby unraveling an important molecule underlying the morphogenesis of the shell field. Moreover, it would be intriguing to explore whether these processes are mediated by common cellular mechanisms. Theoretically, a presumptive contractile force toward the central area of the shell field, as well as toward the internal of the embryo, would underpin all three processes.

As mentioned above, actomyosin can participate in cell specifications in a particular developmental process (e.g., Kim et al., 2015). Therefore, one should not assume the roles of NM II to be restricted to morphogenetic processes such as invagination. Thus, we investigated the expression of several pSF genes to explore whether NM II participates in the specification of shell field cells. Despite the considerable changes in expression patterns after blebbistatin treatments, we did not detect evident variations in the expression levels of pSF genes when shell field morphogenesis was completed (8 hpf). This result seemed to indicate that the actomyosin networks were not involved in the regulation of these genes (thus the specification of related cells). However, it is too soon to reach this conclusion since the genes we investigated were still limited and unrecognized gene expression changes could be involved. Investigations of additional pSF genes are required to assess the roles of actomyosin networks in the cell specifications of the shell field.

### Potential Prevalence of the Involvement of Actomyosin Networks in the Morphogenesis of Molluskan Shell Fields

Given the prevalence of shell field invagination in mollusks (Kniprath, 1981) and the roles of actomyosin networks in *L. goshimai*, it would be intriguing to ask whether actomyosin networks would also mediate shell field invagination in other molluskan lineages. If this were true, one would expect to detect aggregations of F-actin in the shell field in various molluskan lineages. However, although investigations of F-actin in molluskan embryos/larvae have been extensive (Wanninger et al., 1999; Wanninger and Haszprunar, 2002; Dyachuk and Odintsova, 2009; Kristof et al., 2016; Battonyai et al., 2018; Yurchenko et al., 2018), F-actin staining was mainly used to detect muscular tissues in late larvae (e.g., veliger). Even though early embryos/larvae are investigated occasionally (Wanninger et al., 1999), the very strong F-actin signals in the muscular cells may have affected the detection of F-actin in nonmuscular tissues. To the best of our knowledge, two studies investigated F-actin distributions without focusing on the muscular system. In the bivalve *Saccostrea kegaki*, Kin et al. did not reveal F-actin aggregation in the 48-cell embryo, although the shell field started its early morphogenesis at this stage (X cells that would comprise the shell field emerged, and they showed a trend of invagination) (Kin et al., 2009). Thus, this result seems to not support the role of actomyosin networks in the shell field morphogenesis of *Saccostrea*. However, the lack of F-actin aggregation in *Saccostrea* could be interpreted as indicating that the embryo was at too early a stage of shell field morphogenesis and that aggregation might be expected at later stages. In the gastropod *Ilyanassa obsoleta*, in contrast, strong F-actin staining could be observed in the margin of the shell field (the newly formed mantle) in the four-day-old embryo (Johnson et al., 2019), when the shell plate is at its early phase of growth. This distribution pattern of F-actin in *Ilyanassa* embryos is highly similar to what we observed in the early trochophores of *L. goshimai* (Figure 2), suggesting the common involvement of actomyosin networks in shell development in the two species. Nevertheless, since shell field morphogenesis has been completed in four-day-old *Ilyanassa* embryos (in particular, invagination has finished), it is also possible that the strong F-actin signals in *Ilyanassa* reflect the developing muscular tissues in the mantle [as seen in many mollusks (Audino et al., 2015)]. Altogether, current data do not simply support or deny the widespread involvement of actomyosin networks in molluskan shell field morphogenesis. Investigating F-actin in the developing shell field in multiple molluskan species (especially during invagination) is necessary to explore the prevalence of this mechanism.

### NM II Is Crucial for the Formation of Larval Shell Plates

We found that although the morphology of the shell field was only moderately inhibited after short-term NM II pulses, such treatments were sufficient to prevent the initial formation of the shell plate (10 hpf, Figure 4), followed by the complete loss of the shell plate in the veliger larvae (24 hpf, Figure 3). We suspected that this might be due to the loss of the capacity of the larvae to synthesize shell materials. However, the relatively unchanged expression level of *tyrosinase*, a potential shell biogenesis gene, indicated that the capacity to synthesize the shell materials was likely unaffected. This idea was further supported by the incomplete but evident residual shell pieces in manipulated 10-hpf trochophore larvae (Figure 4). Despite being seriously reduced, these residual shell pieces suggested that the shell materials could be synthesized, secreted into extracellular spaces, and finally form the shell (albeit incomplete). We propose two possibilities to explain the lack of larval shell plates after the blebbistatin treatments. First, the capacity to secrete shell materials may have been inhibited by the treatments. NM II has been revealed to function in intracellular trafficking and secretion (Miklavc et al., 2012; Bedi et al., 2017). It is possible that inhibition of NM II function results in an impaired secreting capacity of related cells, which may not be able to meet the requirement to form a complete shell plate but can only catalyze the formation of a residual shell piece. Alternatively, it is possible that the formation of a complete shell plate requires the coordination of different cells, which relies on a particular organization of the shell field. Blebbistatin treatments caused changes in the organization of the shell field (despite the possibly unaffected cell fates), which might destroy the coordination of different cells and thus affect the formation of the shell plate. This idea is partially supported by the highly regular organization of the cells inside the shell field immediately before shell secretion (Kniprath, 1981). Previous studies have also revealed that the shell materials secreted by different cells are transferred to the extracellular space, where they are catalyzed to crosslink with each other to form the shell plate (Kniprath, 1980; Bielefeld and Becker, 1991). It is possible that when the shell field became disorganized after the blebbistatin treatments, although the shell materials were secreted and translocated to extracellular regions, together they could not generate a correct arrangement to form a complete shell plate. This proposal is consistent with previous observations of manipulated embryos that frequently develop birefringent materials but do not show complete shell plates (Clement, 1962; Boring and July 1989; Lambert and Nagy, 2001; Henry et al., 2017).

Another notable fact is that short-term blebbistatin pulses during the morphogenesis of the shell field (in as short as 1 hour) were sufficient to inhibit the formation of larval shell plates in late larvae (24 hpf). Even though the shell field exhibited a trend of restoration when blebbistatin was removed (reflected by the remerged U-shaped expression pattern of pSF genes and malformed but detectable shell pieces at 10 hpf), the capacity to form a shell plate appeared to never recover in the manipulated larvae, potentially because of the misspecification of particular cells (though not detected in the present study), lack of coordination of different cells in the disorganized shell field, or the lack of a structural base from a complete initial shell plate for subsequent shell growth. Further investigations are required to explore the underlying causes. Despite these uncertainties, the present results undoubtedly indicate that the period between 6 and 8 hpf is crucial for shell field development, consistent with the time window showing aggregations of F-actin and that NM II is crucial for larval shell development.

## Data Availability

The original contributions presented in the study are included in the article/Supplementary Material, further inquiries can be directed to the corresponding author.

## References

[B1] Agarwal P. Zaidel-Bar R. (2019). Principles of Actomyosin Regulation *In Vivo* . Trends Cel Biol. 29, 150–163. 10.1016/j.tcb.2018.09.006 30385150

[B2] Audino J. A. Marian J. E. A. R. Wanninger A. Lopes S. G. B. C. (2015). Mantle Margin Morphogenesis in *Nodipecten Nodosus* (Mollusca: Bivalvia): New Insights into the Development and the Roles of Bivalve Pallial Folds. BMC Dev. Biol. 15, 22. 10.1186/s12861-015-0074-9 26017922 PMC4445998

[B3] Battonyai I. Voronezhskaya E. E. Obukhova A. Horváth R. Nezlin L. P. Elekes K. (2018). Neuronal Development in the Larvae of the Invasive BiofoulerDreissena polymorpha(Mollusca: Bivalvia), with Special Attention to Sensory Elements and Swimming Behavior. Biol. Bull. 234, 192–206. 10.1086/698511 29949436

[B4] Bedi D. Dennis J. C. Morrison E. E. Braden T. D. Judd R. L. (2017). Regulation of Intracellular Trafficking and Secretion of Adiponectin by Myosin II. Biochem. Biophysical Res. Commun. 490, 202–208. 10.1016/j.bbrc.2017.06.021 28606474

[B5] Bielefeld U. Becker W. (1991). Embryonic Development of the Shell in biomphalaria Glabrata (Say). Int. J. Dev. Biol. 35, 121–131. 1768600

[B6] Boring L. July A. (1989). Cell-cell Interactions Determine the Dorsoventral axis in Embryos of an Equally Cleaving Opisthobranch Mollusc. Dev. Biol. 136, 239–253. 10.1016/0012-1606(89)90145-0 2806721

[B7] Clement A. C. (1962). Development ofIlyanassa Following Removal of the D Macromere at Successive Cleavage Stages. J. Exp. Zool. 149, 193–215. 10.1002/jez.1401490304

[B8] Dong B. Deng W. Jiang D. (2011). Distinct Cytoskeleton Populations and Extensive Crosstalk Control *Ciona* Notochord Tubulogenesis. Development 138, 1631–1641. 10.1242/dev.057208 21427145

[B9] Dyachuk V. Odintsova N. (2009). Development of the Larval Muscle System in the Mussel *Mytilus trossulus* (Mollusca, Bivalvia). Dev. Growth Differ. 51, 69–79. 10.1111/j.1440-169x.2008.01081.x 19207179

[B10] Eyster L. S. (1983). Ultrastructure of Early Embryonic Shell Formation in the Opisthobranch Gastropod *Aeolidia papillosa* . Biol. Bull. 165, 394–408. 10.2307/1541204 28368232

[B11] Fritsch M. Wollesen T. Wanninger A. (2016). Hox and ParaHox Gene Expression in Early Body Plan Patterning of Polyplacophoran Mollusks. J. Exp. Zool. (Mol. Dev. Evol. 326, 89–104. 10.1002/jez.b.22671 PMC4949717 27098677

[B12] Heisenberg C.-P. Bellaïche Y. (2013). Forces in Tissue Morphogenesis and Patterning. Cell 153, 948–962. 10.1016/j.cell.2013.05.008 23706734

[B13] Heissler S. M. Manstein D. J. (2013). Nonmuscle Myosin-2: Mix and Match. Cell. Mol. Life Sci. 70, 1–21. 10.1007/s00018-012-1002-9 22565821 PMC3535348

[B14] Henry J. Q. Lyons D. C. Perry K. J. Osborne C. C. (2017). Establishment and Activity of the D Quadrant Organizer in the marine Gastropod *Crepidula Fornicata* . Dev. Biol. 431, 282–296. 10.1016/j.ydbio.2017.09.003 28887017

[B15] Hohagen J. Jackson D. J. (2013). An Ancient Process in a Modern Mollusc: Early Development of the Shell in *Lymnaea stagnalis* . BMC Dev. Biol. 13, 27. 10.1186/1471-213X-13-27 23845038 PMC3728215

[B16] Huan P. Liu G. Wang H. Liu B. (2013). Identification of a Tyrosinase Gene Potentially Involved in Early Larval Shell Biogenesis of the Pacific Oyster *Crassostrea gigas* . Dev. Genes Evol. 223, 389–394. 10.1007/s00427-013-0450-z 23897397

[B17] Huan P. Wang Q. Tan S. Liu B. (2020). Dorsoventral Decoupling of Hox Gene Expression Underpins the Diversification of Molluscs. Proc. Natl. Acad. Sci. USA 117, 503–512. 10.1073/pnas.1907328117 31871200 PMC6955316

[B18] Jacinto A. Baum B. (2003). Actin in Development. Mech. Dev. 120, 1337–1349. 10.1016/j.mod.2003.06.006 14623442

[B19] Johnson A. B. Fogel N. S. Lambert J. D. (2019). Growth and Morphogenesis of the Gastropod Shell. Proc. Natl. Acad. Sci. U S A. 116, 6878–6883. 10.1073/pnas.1816089116 30867292 PMC6452709

[B20] Kim K. Ossipova O. Sokol S. Y. (2015). Neural Crest Specification by Inhibition of the ROCK/Myosin II Pathway. Stem Cells 33, 674–685. 10.1002/stem.1877 25346532 PMC4428345

[B21] Kin K. Kakoi S. Wada H. (2009). A Novel Role for *Dpp* in the Shaping of Bivalve Shells Revealed in a Conserved Molluscan Developmental Program. Dev. Biol. 329, 152–166. 10.1016/j.ydbio.2009.01.021 19382296

[B22] Kniprath E. (1980). Larval Development of the Shell and the Shell Gland inMytilus (Bivalvia). Wilhelm Roux' Archiv 188, 201–204. 10.1007/bf00849049 28305757

[B23] Kniprath E. (1981). Ontogeny of the Molluscan Shell Field: a Review. Zool Scripta 10, 61–79. 10.1111/j.1463-6409.1981.tb00485.x

[B24] Koop D. Richards G. S. Wanninger A. Gunter H. M. Degnan B. M. (2007). The Role of MAPK Signaling in Patterning and Establishing Axial Symmetry in the Gastropod *Haliotis Asinina* . Dev. Biol. 311, 200–212. 10.1016/j.ydbio.2007.08.035 17916345

[B25] Kristof A. Oliveira A. L. Kolbin K. G. Wanninger A. (2016). Neuromuscular Development in Patellogastropoda (Mollusca: Gastropoda) and its Importance for Reconstructing Ancestral Gastropod Bodyplan Features. J. Zoolog. Syst. Evol. Res. 54, 22–39. 10.1111/jzs.12112 PMC4747121 26869747

[B26] Lambert J. D. Nagy L. M. (2001). MAPK Signaling by the D Quadrant Embryonic Organizer of the Mollusc Ilyanassa Obsoleta. Development (Cambridge, England) 128, 45–56. 10.1242/dev.128.1.45 11092810

[B27] Lyons D. C. Weisblat D. A. (2009). D Quadrant Specification in the Leech *Helobdella*: Actomyosin Contractility Controls the Unequal Cleavage of the CD Blastomere. Dev. Biol. 334, 46–58. 10.1016/j.ydbio.2009.07.007 19607823 PMC3077801

[B28] Major R. J. Irvine K. D. (2006). Localization and Requirement for Myosin II at the Dorsal-Ventral Compartment Boundary of theDrosophila wing. Dev. Dyn. 235, 3051–3058. 10.1002/dvdy.20966 17013876

[B29] Miklavc P. Hecht E. Hobi N. Wittekindt O. H. Dietl P. Kranz C. (2012). Actin Coating and Compression of Fused Secretory Vesicles Are Essential for Surfactant Secretion-Aa Role for Rho, Formins and Myosin II. J. Cel Sci 125, 2765–2774. 10.1242/jcs.105262 22427691

[B30] Mouëza M. Gros O. Frenkiel L. (2006). Embryonic Development and Shell Differentiation in *Chione Cancellata* (Bivalvia, Veneridae): an Ultrastructural Analysis. Invertebrate Biol. 125, 21–33. 10.1111/j.1744-7410.2006.00036.x

[B31] Silberfeld T. Gros O. (2006). Embryonic Development of the Tropical Bivalve *Tivela Mactroides* (Born, 1778) (Veneridae: Subfamily Meretricinae): a SEM Study. Cahiers de biologie Mar. 47, 243.

[B32] Straight A. F. Cheung A. Limouze J. Chen I. Westwood N. J. Sellers J. R. (2003). Dissecting Temporal and Spatial Control of Cytokinesis with a Myosin II Inhibitor. Science 299, 1743–1747. 10.1126/science.1081412 12637748

[B33] Tan S. Huan P. Baozhong L. (2018). The Expression Pattern of a Tyrosinase Gene Potentially Involved in Early Larval Shell Biogenesis of the Limpet *Lottia Goshimai* . Mar. Sci. 42, 17–21. 10.11759/hykx2018032002

[B34] Tan S. Huan P. Liu B. (2017). Expression Patterns Indicate that BMP2/4 and Chordin, Not BMP5-8 and Gremlin, Mediate Dorsal-Ventral Patterning in the Mollusk *Crassostrea gigas* . Dev. Genes Evol. 227, 75–84. 10.1007/s00427-016-0570-3 27987051

[B35] Tan S. Huan P. Liu B. (2021). Molluskan Dorsal-Ventral Patterning Relying on BMP2/4 and Chordin Provides Insights into Spiralian Development and Evolution. Mol. Biol. Evol., 1–18. 10.1093/molbev/msab322 34751376 PMC8789067

[B36] Toledo-Jacobo L. Henson J. H. Shuster C. B. (2019). Cytoskeletal Polarization and Cytokinetic Signaling Drives Polar Lobe Formation in Spiralian Embryos. Dev. Biol. 456, 201–211. 10.1016/j.ydbio.2019.08.020 31479647 PMC6925573

[B37] Tomlinson S. G. (1987). Intermediate Stages in the Embryonic Development of the GastropodIlyanassa Obsoleta: a Scanning Electron Microscope Study. Int. J. Invertebrate Reprod. Dev. 12, 253–280. 10.1080/01688170.1987.10510325

[B38] Vicente-Manzanares M. Ma X. Adelstein R. S. Horwitz A. R. (2009). Non-muscle Myosin II Takes centre Stage in Cell Adhesion and Migration. Nat. Rev. Mol. Cel Biol 10, 778–790. 10.1038/nrm2786 PMC2834236 19851336

[B39] Wanninger A. Haszprunar G. (2002). Muscle Development inAntalis Entalis (Mollusca, Scaphopoda) and its Significance for Scaphopod Relationships. J. Morphol. 254, 53–64. 10.1002/jmor.10004 12219343

[B40] Wanninger A. Haszprunar G. (2001). The Expression of an Engrailed Protein during Embryonic Shell Formation of the Tusk-Shell, *Antalis Entalis* (Mollusca, Scaphopoda). Evol. Dev. 3, 312–321. 10.1046/j.1525-142x.2001.01034.x 11710763

[B41] Wanninger A. Ruthensteiner B. Lobenwein S. Salvenmoser W. Dictus W. J. A. G. Haszprunar G. (1999). Development of the Musculature in the Limpet *Patella* (Mollusca, Patellogastropoda). Dev. Genes Evol. 209, 226–238. 10.1007/s004270050247 10079366

[B42] Yang W. Huan P. Liu B. (2020). Early Shell Field Morphogenesis of a Patellogastropod Mollusk Predominantly Relies on Cell Movement and F-Actin Dynamics. BMC Dev. Biol. 20, 18. 10.1186/s12861-020-00223-3 32814562 PMC7439683

[B43] Yurchenko O. V. Skiteva O. I. Voronezhskaya E. E. Dyachuk V. A. (2018). Nervous System Development in the Pacific Oyster, *Crassostrea gigas* (Mollusca: Bivalvia). Front. Zool 15, 10–21. 10.1186/s12983-018-0259-8 29681988 PMC5896133

