# Supplementary material for: Nonmuscle Myosin II is Required for Larval Shell Formation in a Patellogastropod
Source: Front Cell Dev Biol. 2022 Feb 3;10:813741. doi: 10.3389/fcell.2022.813741 (PMC8851382; doi:10.3389/fcell.2022.813741)
Supplement: Supplementary file 1 [file DataSheet1.pdf]

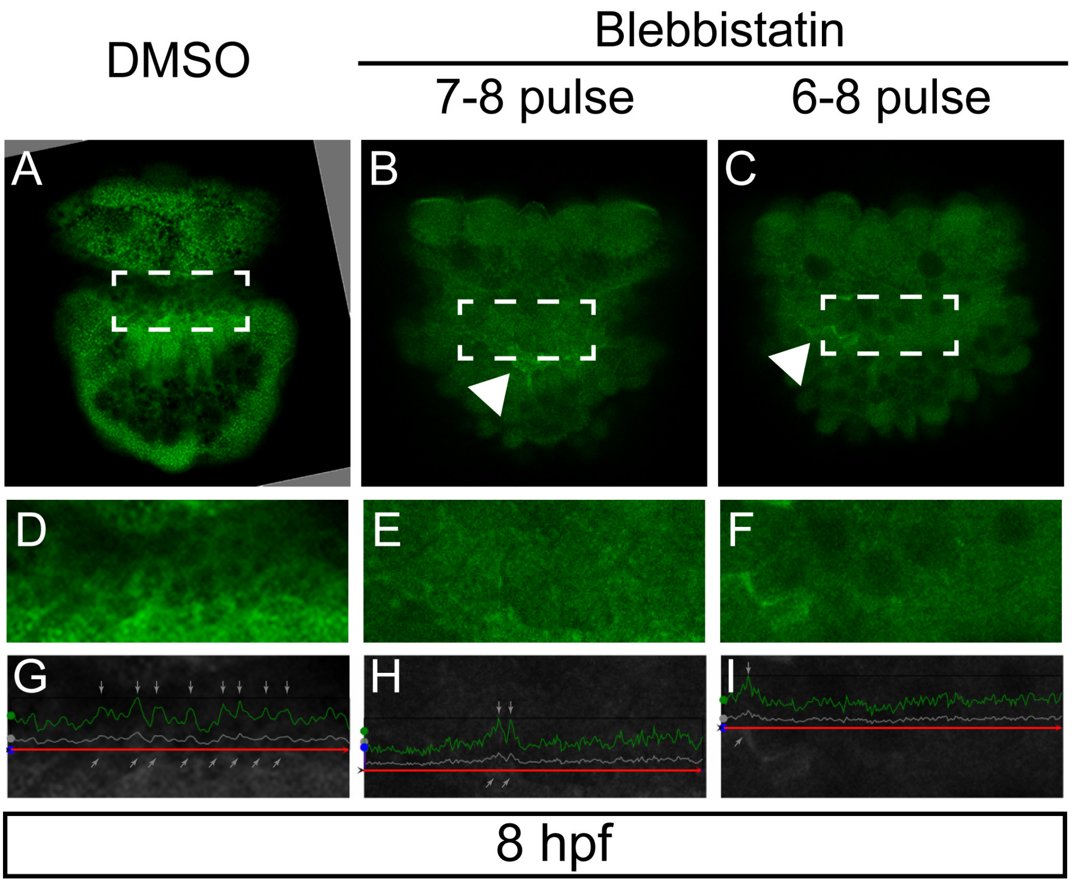

Figure S1 Distributions of pNM II at 8 hpf after two types of blebbistatin pulses and the linear intensity profiles. Panels **A-C** show dorsal views of 8-hpf embryos anterior to the top, which are also presented in Fig. 5. Panels **D-E** and **G-I** show magnified images of the regions labeled in **A-C**, which are colored and grayscale versions, respectively. Linear intensity profiles are provided in **G-I** to show quantitative information for the signals. In each panel, the fluorescence intensities along the red line are provided (the green curved line shown above, measured by Zen microimaging software (Zeiss, version 3.4)). Although staining could also be detected in cytoplasmic regions (see discussion of the staining in the text), the cortical region of a given cell (gray arrows in **G**) showed higher fluorescence intensities than the cytoplasmic region in control embryos. This could also be observed in the manipulated embryos showing residual pNM II signals in cortical regions (gray arrows in **H** and **I**, compare them with the F-actin distributions shown in Fig. 5I and J).

Table S1. Primers used in the study

Gene	GenBank Accession No.	Primers (5'-3')
bmp2/4	MN528131	forward: ACCAGAAGCAAATTCCTCAAGT<br>reverse: CCCTCTACTACCATATCTTGATAG
gata2/3	MN528140	forward: CCATCCGAAGACATAAGTGACA<br>reverse: ATGATAGAGACCACAGGCGTTA
hox1	MK637065	forward: TCGGCTAATGGAACCTGTATG<br>reverse: GTGTATCATGACACATGGCTAA
engrailed	MN326439	forward: TTTATCAATCAAGAGGGAGGTCAG<br>reverse: TGTCCATACTGTCACTATCGTCTA
tyrosinase	OL438768	forward: GAAGCAGGATACAACGATGACT<br>reverse: TCCGGTATGAAGAGGTAGGTTA
